# Processed *vs.* Non-Processed Biowastes for Agriculture: Effects of Post-Harvest Tomato Plants and Biochar on Radish Growth, Chlorophyll Content and Protein Production

**DOI:** 10.3390/ijms16048826

**Published:** 2015-04-21

**Authors:** Chiara Mozzetti Monterumici, Daniele Rosso, Enzo Montoneri, Marco Ginepro, Andrea Baglieri, Etelvino Henrique Novotny, Witold Kwapinski, Michèle Negre

**Affiliations:** 1 Dipartimento di Scienze Agrarie, Forestali e Alimentari, Università di Torino, Largo P. Braccini 2, I-10095 Grugliasco, Italy; E-Mail: chiara.mozzetti@unito.it; 2 ACEA Pinerolese Industriale SpA, Via Vigone 42, I-10064 Pinerolo, Italy; E-Mail: dage@libero.it; 3 Biowaste Processing, Via XXIV Maggio 25, I-37126 Verona, Italy; E-Mail: enzo.montoneri@gmail.com; 4 Dipartimento di Chimica, Università di Torino, Via Giuria 7, I-10125 Torino, Italy; E-Mail: marco.ginepro@unito.it; 5 Dipartimento di Agricoltura, Alimentazione e Ambiente, Università di Catania, Via S. Sofia 98, I-95123 Catania, Italy; E-Mail: abaglie@unict.it; 6 Embrapa Solos, Rua Jardim Botânico, 1024, CEP-22460-000 Rio de Janeiro, RJ, Brazil; E-Mail: etelvino.novotny@gmail.com; 7 Chemical and Environmental Science Department, University of Limerick, Castletroy, Limerick, Ireland; E-Mail: witold.kwapinski@ul.ie

**Keywords:** radish, biochar, post harvest tomato plants, plant growth, chlorophyll content, N assimilation

## Abstract

The aim of this work was to address the issue of processed *vs.* non-processed biowastes for agriculture, by comparing materials widely differing for the amount of process energy consumption. Thus, residual post harvest tomato plants (TP), the TP hydrolysates obtained at pH 13 and 60 °C, and two known biochar products obtained by 650 °C pyrolysis were prepared. All products were characterized and used in a cultivation of radish plants. The chemical composition and molecular nature of the materials was investigated by solid state ^13^C NMR spectrometry, elemental analysis and potentiometric titration. The plants were analysed for growth and content of chlorophyll, carotenoids and soluble proteins. The results show that the TP and the alkaline hydrolysates contain lignin, hemicellulose, protein, peptide and/or amino acids moieties, and several mineral elements. The biochar samples contain also similar mineral elements, but the organic fraction is characterized mainly by fused aromatic rings. All materials had a positive effect on radish growth, mainly on the diameter of roots. The best performances in terms of plant growth were given by miscanthus originated biochar and TP. The most significant effect was the enhancement of soluble protein content in the plants treated with the lowest energy consumption non processed TP. The significance of these findings for agriculture and the environment is discussed.

## 1. Introduction

There are several ways nowadays to treat biowaste in order to obtain products for use in agriculture. These are mainly anaerobic [1] and aerobic [2] fermentation to yield digestate and compost, respectively, and pyrolysis to biochar [3]. These products have been demonstrated to promote plant growth. Humic substances (HS) present in soil and natural waters are also known to consistently increase plant growth [4,5]. Unfortunately, exploitation of HS for real agriculture practices is not sustainable economically and environmentally, due to the low concentration and depletion from soil and water. Searching for more sustainable HS-like sources, soluble substances have been obtained by hydrolysis of composted bio-wastes. These substances have been demonstrated to bear structure similarities with HS and to be very effective as plant growth and productivity promoters [6].

The use of the above materials implies sustaining biowaste processing costs. Biochar is likely to be an expensive input, compared to organic amendments such as compost [7]. Very recently, Baglieri *et al.* [8] tested non-composted agriculture residues in pot cultivation of bean plants. They used post-harvest tomato plants (TP) and soluble (SOL) and insoluble (INS) products obtained by TP hydrolysis at 60 °C in addition to a commercial substrate for the growth of bean plants. The products were compared for effects on biomass production, chlorophyll content and N assimilation. The insoluble and soluble substances sourced by alkaline hydrolysis of the tomato plant powder exhibit strong effects, mainly the increase of nitrogen assimilation typical of biostimulants.

Proving the observed effects general for other plant species would add a valuable argument for using TP and/or its hydrolysis products in agriculture. At the same time, comparing the non-processed TP with other materials obtained under different processing costs, for the added value as plant growth promoters, would contribute arguments addressing the issue of biowaste processing costs *vs.* benefits in real agriculture practices.

The present work originated from the cooperation of two research groups, respectively located in Italy and Ireland. This cooperation was carried out within the framework of the COST-European Cooperation in Science and Technology EUBis Action TD1203. The two groups had in common the objective of valorizing biowastes as a source of added value products. However, they used two different biowaste processing technologies. The Italian research groups applied a low temperature hydrolysis process to convert biowastes to soluble products for diversified applications [9]. The Irish group applied thermal processes [10] such as the pyrolysis of biomass in the absence of oxygen, to produce a combustible gas mixture and biochar-C rich solid residue. Thus, materials originating from different sources, and obtained under widely different processing costs, such as cutting and grinding only (TP), and adding to mechanical pretreatment low temperature hydrolysis (SOL and INS) or high temperature pyrolysis (biochar), were available from the two groups. This circumstance offered the opportunity to report, for the first time, in the present work, the direct comparison of a non-processed agriculture residue, such as TP, with products obtained from processed materials, such as the SOL and INS obtained by hydrolysis at 60 °C [8] and biochar obtained by 650 °C pyrolysis [10,11] of poultry manure (BIOP), produced at industrial scale, and miscanthus (BIOM), produced in the laboratory.

Several important economic and environmental aspects were also connected with investigating the above specific materials. First, worldwide tomato production [12] is estimated at 150 million ton per year. Thus, abundance of post-harvest residues from this culture is available. For viable exploitation, the material geographical concentration is however more important than the total amount. For the present work, TP were sourced from a location where cultivation is practiced intensively by 4168 farms in open fields and green house installations are distributed over 9156 ha area. These farms are located within 30 km of the center of this area. They produce 25,000 ton year^−1^ post-harvest horticulture residual dry matter, containing 20,000 ton organic matter [13]. The cultivation residues are currently burned at each farm site in the open field. Thus, a large amount of agriculture residues concentrated over a relatively small area is produced in this part of Italy and needs more eco-friendly disposal practices. These geographic and production features guarantee the availability of a concentrated potentially cost effective lignocellulosic waste feedstock. They make the above chosen location and its horticulture residues rather appealing for the installation of a biorefinery which is not critically burdened with feed material collection and transportation costs. The work performed by Baglieri *et al.* [8] is a first step promoting this scenario. On the other hand, biochar is a solid material obtained from the carbonisation of biomass that is added to soils with the intention to improve soil functions and to reduce greenhouse gas emissions from the decomposition of biomass. It includes a spectrum of materials with certain characteristics, depending on process conditions of production, and on the feedstock that is used. Any agricultural waste products can be converted into biochar including forestry, crop waste, and animal manures. The choice of feedstock significantly impacts the biochar product structure and composition, and its potential application. Depending on the production parameters, more than 50% of the organic material’s carbon may be sequestered in a non-volative form in the biochar. When the biochar is used as a soil amendment, a significant proportion of the recalcitrant biochar carbon can resist degradation for hundreds to even thousands of years, thus creating stable carbon pools. Other significant benefits of biochar include improved soil fertility, crop productivity, water retention in certain types of soils, the reduced need for additional fertilizer usage, and reduced nutrient leaching. In addition, bio-fuels and process heat can be created during the production process. The BIOP [11,14,15] and BIOM [10] materials are among the most investigated biochar products sourced from animal and vegetable feedstock crop.

For the purpose of the present work, the above products were tested using radish as probe cultivation species, due to its rapid growth cycle. The products were compared for their effects on the plant biomass production, chlorophyll content and N assimilation. For the types of investigated products, the present work carries out two types of comparison: *i.e.*, (i) the comparison of biowaste processing *vs.* non-processing; and (ii) the comparison of different products. The comparison of TP and its INS and SOL hydrolysates allows an appreciation of the effects of the non-processed biowaste *vs.* the same biowaste after processing. In essence it allows the assessment of whether, in the specific case of TP, it is worth to process the biowaste or not. The comparison of TP, INS and SOL *vs.* the two BIOM and BIOP biochars is mainly a comparison of products from different sourcing biowastes and processes. The TP, INS and SOL are very new materials which have been tested only for their effects on bean plants [8]. On the other hand, BIOM and BIOP are well know materials which could perform as reference materials for rating the value of the new TP, INS and SOL products.

## 2. Results and Discussion

### 2.1. Chemical Nature of Investigated Products

Table 1 reports the chemical characterisation of the investigated products. The total C, N and P content of the tomato plant powder (TP) reflects the composition of vegetal material while after the hydrolysis process, these elements are distributed more in the soluble (SOL) than in the insoluble (INS) fraction. There are large differences between the two biochars: BIOP composition is close to that of the other materials, except for the P content which is much higher, reflecting the high content of this element in the poultry litter. In contrast, BIOM is poor in N and P but contains 85% C. The nitric-N content of TP is about one tenth of the total N content while the nitric-N content of all materials is below or close to the detection limit. More than 20% of P is available in all materials but SOL, suggesting that, in this last case, P is strongly bound to the organic material. In contrast, all the P contained in BIOM is in available form.

**Table 1:** Chemical composition of the investigated products.

	Ash, g/Kg	pH	Organic C, g/Kg	Total N, g/Kg	Nitric N, g/Kg	C/N	Total P, g/Kg	Available P, g/Kg
BIOM	94	10.1	851.0 ± 2.0	5.5 ± 0.6	<0.05	155	2.50 ± 0.03	2.49 ± 0.08
BIOP	435	10.2	486 ± 1.7	39.0 ± 0.7	<0.05	12.4	24.6 ± 0.50	7.49 ± 0.35
TP	202	7.6	364.4 ± 1.6	35.1 ± 0.5	3.9	10.4	3.32 ± 0.25	0.81 ± 0.01
SOL	232	9.4	473.0 ± 1.0	65.2 ± 0.6	0.05	7.3	9.76 ± 0.32	0.25 ± 0.02
INS	369	7.3	288.3 ± 0.9	25.2 ± 0.4	<0.05	11.4	3.28 ± 0.29	0.87 ± 0.01
substrate	454	6.4	248 ± 1.3	9.0 ± 0.3	0.1	27.5	2.56 ± 0.25	0.53 ± 0.01

The tomato plant powder and the two hydrolysis products contain organic matter as major component. The chemical nature of the organic matter in TP was first analyzed according to a procedure expected to separate the major biomass proximates on the basis of the components solubility in benzene/ethanol and in mineral acid at different temperatures [8]. The results indicated that TP contains 11.9% *w*/*w* lipids and non-polar compounds, 44% *w*/*w* hemicelluloses and proteins, 15.5% *w*/*w* cellulose and 28.5% *w*/*w* lignin. Further details of the nature of the organic matter in the TP, SOL and INS materials were obtained by ^13^C NMR spectroscopy.

**Figure 1:**
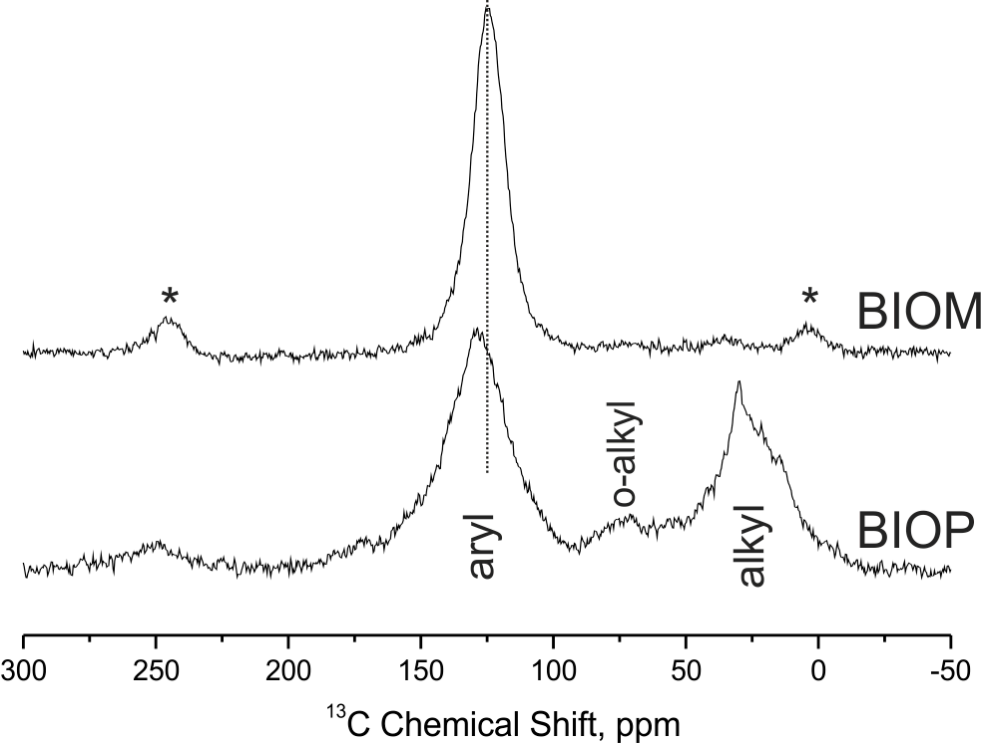
^13^C NMR solid state spectra of poultry litter and miscanthus biochar samples. The symbols * indicate the spinning sidebands and the vertical dot line show the up field shift of the aryl signal.

**Figure 2:**
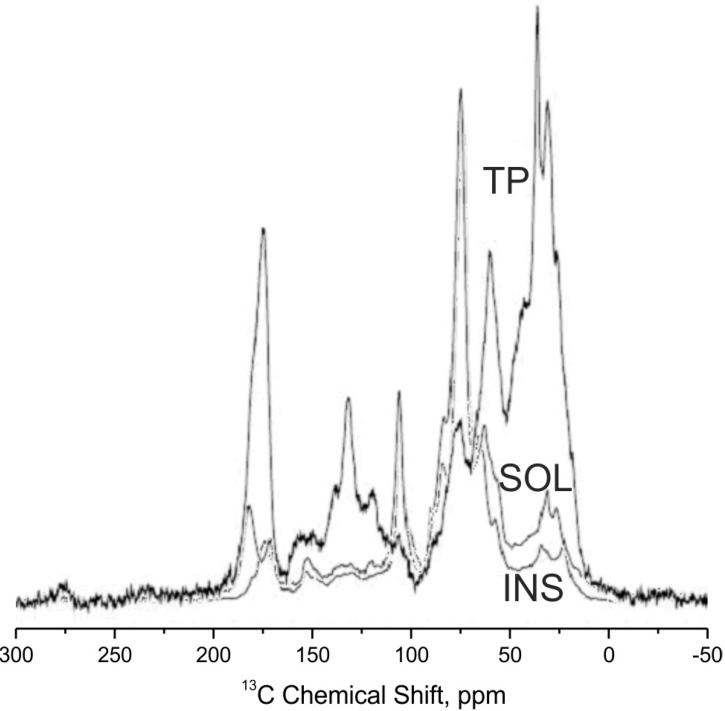
^13^C NMR solid state spectra of TP, SOL and INS.

Figure 1 and Figure 2 report the corresponding ^13^C solid-state NMR spectra recorded under different experimental conditions (see Section 3.5). It is evident that the biochar samples are much richer in aromatic C. The NMR spectra of the BIOM and BIOP samples (Figure 1) show the symmetrical aromatic resonances that are characteristic of chars [10,11]. In case of BIOM there is only a single, featureless and narrow symmetrical aryl signal, while BIOP shows a broader aryl signal and also aliphatic residues (alkyl and O-alkyl regions). Moreover, the aryl signal for the BIOM sample is further upfield shifted (lower chemical shift around 124 ppm), than that of the BIOP sample, whose signal is centred at 129 ppm. This indicates higher aromatic ring condensation [16,17] for the BIOP sample. All these features, *i.e.*, residual O-alkyl and alkyl, and broader asymmetrical aryl signal, probably due to signal overlapping of complex residual aromatics groups, indicate that BIOP is less carbonised material than BIOM. Due the aromatic condensation, the BIOM and BIOP materials are H-poor and so, some ^13^C are far from ^1^H, resulting in very poor polarisation. This results in a selective loss of signal for condensed aromatic C, while protonated C groups are overestimated. Thus the quantitative estimate of the relative C types and functional groups composition for the BIOM and BIOP samples are not reported.

Figure 2 and Table 2 show that the ^13^C spectra of the tomato plant residues and its hydrolysates are consistent with the presence of aliphatic, methoxy (OMe), ammine, alkoxy (OR), anomeric (OCO), aromatic, phenoxy and phenol (PhOY, Y = Ph, R, H), amide and carboxylic acid (COX, X = N or H), ketone (C=O) C. Anomeric (OCO) and alkoxy (OR) functional groups are contributed by the cellulose and hemicelluloses organic fraction and the aromatic (Ph) and phenoxy (PhOX) moieties by native lignin. These moieties are still present in the SOL and INS hydrolysates, but with different distribution. The SOL, isolated as the retentate of ultrafiltration through 5 kD cut off membranes (see Experimental Section), and more so the INS, in reason of its insolubility, are still polymeric compounds. The C types and functional groups characterizing the organic matter of the hydrolysates are the likely memory of the pristine lignocellulosic proximates. Compared to the parent TP, SOL is richer in lignin-like matter, whereas INS is richer in cellulose. In addition to the ^13^C NMR data, the potentiometric titration of the SOL has allowed to breakdown the 14% COX carbon fraction into 12% carboxylate and 2% amide C. Consequently it has been possible to calculate the distribution of the total SOL N content in Table 2 between amide and ammine functional groups. The calculation yields 9.4% C bonded to amine groups and nearly none as methoxy groups. The presence of both carboxylic and ammine groups is a likely indication of protein, peptide or amino acid residues composing the SOL together with lignin and hemicellulose moieities. The same breakdown of COX and total OME and NR functional groups could not be obtained for TP and INS, since both are insoluble and cannot be reliably titrated in homogeneous solution.

The difference in the organic matter nature is consistent with the different production processes. In essence, the low temperature hydrolysis SOL and INS products keep the memory of the pristine TP, although exhibit significant differences in the distribution of the organic fraction, and in its composition and solubility. On the contrary, the high temperature pyrolytic treatment induces great chemical and physico-chemical changes in the pristine biomass. It converts the saccharide and aliphatic matter of the pristine miscanthus and poultry litter into gas and liquid hydrocarbons, and the lignin matter into biochar made of fused aromatic rings only. Generally, lower yields of biochar products are obtained upon increasing the pyrolysis temperature.

The investigated products were also analyzed for the total and soluble silicium and metal content. Table 3 shows that the total metal composition of BIOM and BIOP reflects the different origin of the starting materials: BIOP is much richer in all metals, likely coming from dejections of the animals. The very high content in Cu and Zn is consistent with addition of these elements in feed. The much lower concentration of all total metals in BIOM cannot be attributed only to the vegetal origin of this biochar. It could be the result of the high preparation temperature causing a partial loss of some salts. Consistently, the untreated TP vegetable matter has much higher concentrations of all total metals. The TP hydrolysis process then provokes a further repartition of the total metals and silicium between SOL and INS. The high concentrations of Cu and Zn in SOL suggest that this material, by virtue of its functional acid and basic groups is able to strongly link these metal cations.

**Table 2:** C types and functional groups ^a^ distribution as mole % of total organic C for the tomato plant powder (TP), and the SOL and INS hydrolysates.

	Al	OMe + NR	OR	OCO	Ph	PhOY	COX	CO
TP	14.34	7.22	49.60	11.62	6.89	3.44	6.28	0.61
SOL	47.38	9.39	10.39	2.19	11.50	3.81	14.37	0.97
INS	5.00	7.97	58.98	13.19	7.00	3.66	2.97	1.22

^a^ Aliphatic (Al), methoxy (OMe), ammine (NR), alkoxy (RO), anomeric (OCO), aromatic (Ph), phenoxy and phenol (PhOY, Y = Ph, R, H), amide and carboxylic acid (COX, X = N or H), ketone (C=O) C.

Table 3 also shows that in all investigated products, about 50% of the total K and Na is water soluble, as expected due to the high solubility of alkaline metal compounds. For the other elements, the amount recovered in solution depends on their solubility. It is always very low (<7%) in the case of the biochars, probably because the mineral elements are present in insoluble oxide form. In contrast, the variety of organic functional groups of TP, SOL and INS likely promotes the complexed forms of the metals, allowing the release of a up to 30% as soluble cations.

**Table 3:** Total (tot) and soluble (sol) mineral elements concentration and sol/tot percentage relative to the pot substrate and the pristine added products.

	Si			K			Mg			Ca			Na			Fe		
	tot	sol	sol/tot	tot	sol	sol/tot	tot	sol	sol/tot	tot	sol	sol/tot	tot	sol	sol/tot	tot	sol	sol/tot
	g/Kg	mg/Kg	%	g/Kg	g/Kg	%	g/Kg	g/Kg	%	g/Kg	g/Kg	%	g/Kg	g/Kg	%	g/Kg	g/Kg	%
BIOM	9.8	110	1.1	18.5	10.1	54.6	1.6	0.06	3.7	2.6	0.06	2.2	1.2	0.33	27.7	0.2	1.3	0.68
BIOP	3.33	16	0.5	78.7	39.3	49.9	21.9	0.67	3.1	44.7	0.44	1.0	14.0	4.57	32.6	3.5	36.7	1.05
TP	9.8	5.5	0.1	33.0	22.3	67.6	4.2	2.59	61.7	46.5	3.06	6.6	2.2	1.35	61.4	3.0	114.4	3.81
SOL	2.2	7.3	0.3	91.5	78.5	85.8	8.0	3.37	42.1	21.0	2.97	14.1	2.4	1.83	76.3	3.3	935.6	28.35
INS	8.5	3.1	0.0	44.9	24.6	54.8	2.7	0.65	24.1	44.1	1.49	3.4	1.5	0.7	46.7	2.5	135.2	5.41
substrate	9.7	6.8	0.1	11.5	3.9	33.9	3.4	0.65	19.3	13.3	1.42	10.7	0.87	0.94	109.3	3.7	19.1	0.52
	**Al**			**Cu**			**Ni**			**Zn**			**Cr**			**Mn**		
	**tot**	**sol**	**sol/tot**	**tot**	**sol**	**sol/tot**	**tot**	**sol**	**sol/tot**	**tot**	**sol**	**sol/tot**	**tot**	**sol**	**sol/tot**	**tot**	**sol**	**sol/tot**
	**g/Kg**	**mg/Kg**	**%**	**g/Kg**	**g/Kg**	**%**	**g/Kg**	**g/Kg**	**%**	**g/Kg**	**g/Kg**	**%**	**g/Kg**	**g/Kg**	**%**	**g/Kg**	**mg/Kg**	**%**
BIOM	0.1	1.5	1.4	10.6	0.1	1.0	0.3	ND	ND	27.5	1.87	6.8	0.7	ND	ND	189	5.5	2.9
BIOP	1.6	7.5	0.5	268.0	7.6	2.8	17.7	0.9	5.3	671.0	13.8	2.1	14.0	0.4	2.9	1704	13.3	0.8
TP	2.7	46.3	1.7	21.0	6.9	32.8	0.1	0.6	525.0	39.0	7.47	19.2	4.0	0.4	9.0	71.7	14.7	20.7
SOL	3.4	311.5	9.2	898.0	262.9	29.3	11.0	7.9	72.1	404.0	119.8	29.6	7.0	2.0	28.9	133	36	27.1
INS	1.7	200.8	11.8	8.0	6.5	81.5	0.5	ND	ND	12.0	4.31	35.9	2.0	0.7	33.5	77.2	4	5.2
substrate	3.5	24.4	0.7	33.8	2.0	5.9	9.8	0.3	3.4	73.5	2.75	3.7	14.5	0.3	1.9	126	7.9	6.2

Values are means calculated over triplicates; standard deviations as % of mean value ranged from 0.2% at 10 g/Kg mean value level to 13% at 1–2 mg/Kg mean value level.

### 2.2. Cultivation Trials

The amount of applied per pot dose of each investigated material and the corresponding amount of each element (total and soluble amount) are reported in Table 4. The applied dose was calculated for each product in order to add to the cultivation pots the same amount of N with each product, *i.e.*, 66 mg N per pot. This was possible for all treatments, except for BIOM. This product has a very low N content compared to the others. Thus, in order to apply the same 66 mg N dose, the total sample weight to apply would amount to 12.5 g per pot. By comparison, the average applied total sample amount for the other products is 1.8 g per pot. Also, the total amount of C applied with the 12.5 g of BIOM would amount to 10.6 g against the average 0.68 g applied with the other products. Under these circumstances, it was decided to apply the BIOM at 1.7 g total sample weight, close to the applied 1.8 g average total sample amount for the other products. In this fashion, the amounts of applied N and C by BIOM addition to the pot substrate were 9.35 mg per pot and 1.35 g per pot, respectively, against 66 N mg per pot and 0.65–0.83 C g per pot applied by the other treatments. Form the economic point of view of the farmers, cost and benefits are more readily appreciated based on product weight rather than on content of elements.

**Figure 3:**
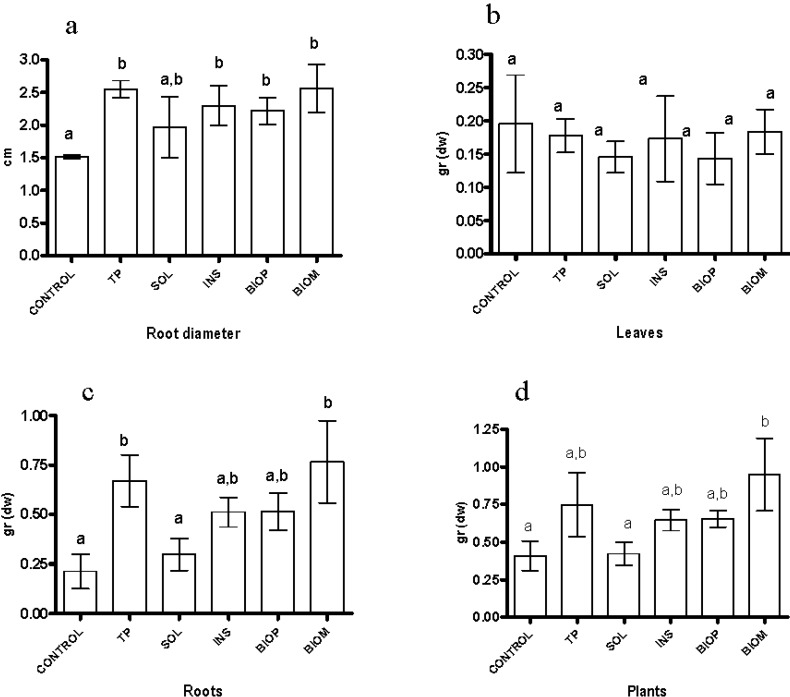
Plant performance indexes *vs.* treatments (**a**) root diameter; (**b**) leaves dry weight; (**c**) roots dry weight; (**d**) whole plant dry weight. Values are the mean of three replications with standard deviation. Letters in columns indicate statistical significance—samples not sharing a letter differ significantly at *p* < 0.05.

**Figure 4:**
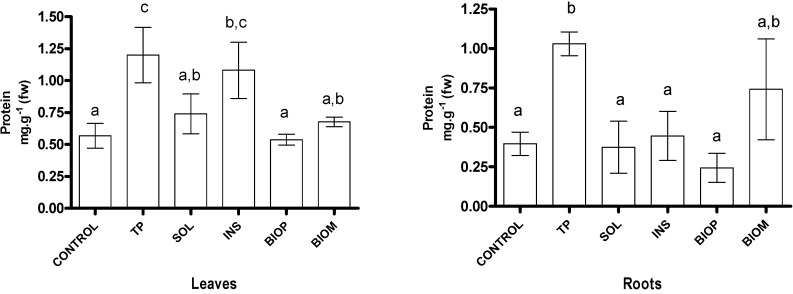
Plant protein content. Values are the mean of three replications with standard deviation. Letters in columns indicate statistical significance—samples not sharing a letter differ significantly at *p* < 0.05.

**Figure 5:**
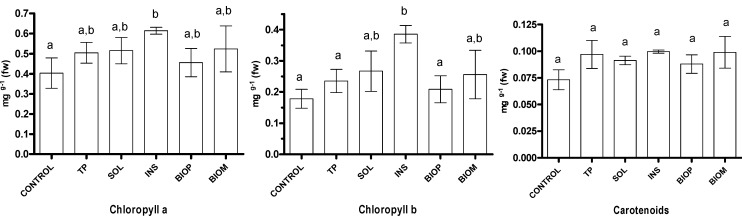
Plant pigment content. Values are the mean of three replications with standard deviation. Letters in columns indicate statistical significance—samples not sharing a letter differ significantly at *p* < 0.05.

From Table 4 data it can be seen that, due to the low application doses, the amount of total and soluble nutrients deriving from the addition of the TP and biochar products was at least one order of magnitude lower than that of the control substrate. Consequently, the positive effects of some of the added products on the plant growth should not be directly related to increase of the nutrients concentration. Figure 3, Figure 4 and Figure 5 report the values measured for the investigated plant parameters for the different treatments. It may be observed that addition of all the tested materials to the growth substrate has a positive or null effect on the measured parameters attesting for their lack of phytotoxicity. All the tested materials but SOL significantly increased the roots size (Figure 3a). In most cases, the average diameter of the roots was close to twice that of the control. No significant differences were observed between the dry weight of the shoots of the plants grown on the treated substrate and that of the control plants (Figure 3b). In contrast, addition of TP and BIOM to the substrate, besides the diameter, significantly increased the dry weight of the roots (Figure 3c). The good performance of BIOM is also reflected on the dry weight of the whole plants (Figure 3d).

Besides promoting the growth of the plants, addition of TP also significantly increased the protein concentration both in roots and leaves. The data in Figure 4 indicate that this material stimulates the uptake of N and the production of amino acids more than the other treatments. On the contrary, for bean plants, Baglieri *et al.* [8] report SOL yielding the highest N uptake, compared to TP and INS. These authors also report that TP does not stimulate the mineralization of nitrogen in the substrate. Therefore, for TP, one can exclude the enhancement of the N mineralizing microbial biomass as a likely cause of the enhanced N uptake by the radish plant. It is more likely that this effect was attained through the enhancement of the enzymatic systems responsible for nitrogen assimilation such as nitrate reductase, glutamine synthetase and glutamate synthase. This could occur via the assimilation of organic nitrogen, as in amino acids, which are known to be source of N for plant nutrition [18]. The process is presumably selective; it depends on the specific protein carrier binding the nutrient ion and carrying it across plant tissue membranes. Thus, the plant ability to pick and choose nutrients from the soil solution is to some extent relatively unaffected by the nutrients concentration in the soil solution [19].

**Table 4:** Amount of total and soluble mineral elements added per pot.

		Si		K		Mg		Ca		Na		Fe	
		tot	sol	tot	sol	tot	sol	tot	sol	tot	sol	tot	sol
	g/pot	mg/pot	μg/pot	mg/pot	mg/pot	mg/pot	mg/pot	mg/pot	mg/pot	mg/pot	mg/pot	mg/pot	μg/pot
BIOM	1.7	16.7	187.0	31.5	17.2	2.8	0.1	4.3	0.1	2.0	0.6	0.3	2.2
BIOP	1.7	5.7	27.2	133.8	66.8	37.2	1.1	76.0	0.7	23.8	7.8	6.0	62.4
TP	1.9	18.6	10.5	62.7	42.4	8.0	4.9	88.4	5.8	4.2	2.6	5.7	217.4
SOL	1.0	2.2	7.3	91.5	78.5	8.0	3.4	21.0	3.0	2.4	1.8	3.3	935.6
INS	2.6	22.1	8.1	116.7	64.0	7.0	1.7	114.7	3.9	3.9	1.8	6.5	351.5
substrate	140.0	1356.6	952.0	1610.0	546.0	471.8	91.0	1863.4	198.8	120.4	131.6	518.0	2674.0
		**Al**		**Cu**		**Ni**		**Zn**		**Cr**		**Mn**	
		**tot**	**sol**	**tot**	**sol**	**tot**	**sol**	**tot**	**sol**	**tot**	**sol**	**tot**	**sol**
	**g/pot**	**mg/pot**	**μg/pot**	**μg/pot**	**μg/pot**	**μg/pot**	**μg/pot**	**μg/pot**	**μg/pot**	**μg/pot**	**μg/pot**	**μg/pot**	**μg/pot**
BIOM	1.7	0.2	2.6	18.0	0.2	0.4	0.0	46.8	3.2	1.1	0.0	321.3	9.4
BIOP	1.7	2.7	12.8	455.6	12.9	30.1	1.6	1140.7	23.5	23.8	0.7	2896.8	22.6
TP	1.9	5.1	88.0	39.9	13.1	0.2	1.2	74.1	14.2	7.6	0.7	135.1	27.9
SOL	1.0	3.4	311.5	898.0	683.5	11.0	20.6	404.0	311.4	7.0	5.3	133.0	93.6
INS	2.6	4.4	522.1	20.8	6.5	1.3	0.7	31.2	4.3	5.2	0.7	200.7	4.0
substrate	140.0	494.2	3416.0	4732.0	280.0	1370.6	46.2	10,290.0	385.0	2030.0	37.8	17,640.0	1106.0

In the results of this work, it is surprising that BIOM yielded, together with TP, significantly the highest increase of both dry weight and diameter of the roots, compared to the control and the other treatments. Indeed BIOM contributes the lowest amounts of N and mineral elements to the pot substrate. In addition, the BIOM organic matter is mainly constituted by fused aromatic rings. On the other hand, the main peculiarity of BIOM is its very high C contribution. The effect on radish of a similar material, a low nutrient content green waste biochar having 78% total C, has already been reported by van Zwieten *et al.* [20]. These authors observed that the above biochar, when applied at about the same dose applied for BIOM in this work, stimulated the increase of radish biomass weight. In contrast the growth of radish was not improved by addition of a green waste biochar containing 36% C, even at 100 t/ha application rate [21].

The other biochar tested (BIOP) originating from poultry litter did not have any effect on the radish biomass. This result is in contrast with those of Chan *et al.* [22] reporting that poultry litter originated biochars increased radish dry matter yield. This discrepancy could be due to the application rate which was about 3.5 t/ha in our experiment while Chan *et al.* added 10 to 100 t/ha biochar, therefore higher nutrients addition. The results obtained on the two biochars point out that the process conditions such as temperature and rate of heating on biochar properties play an important role in their efficacy as amendment. It has been demonstrated [10] that a biochar from miscanthus formed at 400 °C for 10 min inhibited the growth of maize (*Zea mays* L.) while miscanthus biochar formed at 600 °C for 60 min (the same used in this experiment) stimulated the growth of the plant.

The good performances of low nutrients biochar such as BIOM and the green wastes biochar used by van Zwieten *et al.* [20] suggest that biochars do not act as nutrient source but that the microstructure provided by the remaining aromatic C surviving pyrolysis, exposing a large surface area, is presumably capable to behave as good cation exchanger, therefore stimulating the availability of the nutrients and their uptake by the plants. Another study by Hamer *et al.* [23] proposed that biochar could act both by promoting N mineralisation and enhancing microorganism growth.

The concentration of the main photosynthetic pigments in the leaves are reported in Figure 5. The chlorophyll contents in the leaves were promoted by addition of INS, as already observed in the case of bean plants [8]. The analytical investigation on the nature of INS has concluded that it is mostly composed of polysaccharides, which are among the bioactive molecules that can enhance the production of pigments, therefore the photosynthetic activity and the production of carbohydrates. On the other hand, this effect is generally accompanied by a biomass increase [24] which was not significant in our experiments.

### 2.3. Significance of Results for Agriculture and the Environment

In this study the positive effect of addition of crop residues, their hydolyzates and two biochars on plant production has been observed at lower application rate than usually used for organic fertilizers. This is a great advantage from the economical point of view in terms of production, transport, and application of the fertilizers. From the environmental point of view, we can exclude typical issues such as bad odors and the possible leaching of nitrates and heavy metals, when applying the tested materials in the field. This is because the biochars are stable materials, TP is a crop residue for which application in the field is a common practice and the hydrolyzates are stabilized by the chemical process. The low nitric-N content of these products and the low application dose allow excluding any leaching of nitrates. The chemical structure of TP, SOS and INS, investigated by the ^13^C NMR spectroscopy, point out the presence of acidic groups which are known to strongly bind metallic cations. Moreover the low application rate should exclude any problem of soil metal contamination. For use in agriculture, biochar is likely to be an expensive input, more than organic amendments such as compost [7]. By comparison non-processed biowastes, such as TP, available from locations where intensive agriculture is practiced, fall into the negative cost [16] material category. On the other hand, biowaste pyrolysis or fermentation is necessary to cope with the increasing great amount of biowastes produced by the modern society. These processes are run to decrease the biowaste volume and convert biodegradable to stable marketable products such as syngas, bio-oil, biogas and compost. Use of both processed and non-processed biowaste in agriculture allows to recycle organic C and N, and mineral elements back to soil, and hopefully to replace synthetic commercial fertilizers. The choice of the different processed *vs.* non-processed biowastes for agriculture depends on product performance and cost. According to Sohi [7], biochar may have long-term or longer lasting impacts on soil carbon. However, it is the effect over the plant production cycle that has monetary value for farmers. Fortunately, biochar is multifunctional and one aspect, its interactions with plant nutrients, is of immediate relevance to crop production. Mainly, it concerns the direct release of phosphorus and positive interactions with nitrogen. Emphasizing and accentuating these functions offers a promising way forward, for the economic deployment of biochar. Biochar could provide for more efficient transfer of nutrients from a potential biochar feedstock material to the soil. Since nitrogen in biochar feedstock is largely driven out as gases during pyrolysis, the focus is on phosphorus and potassium. Phosphorus and potassium are stable at pyrolysis temperatures, so the elimination of hydrogen, oxygen and some of the carbon (the main constituents of organic matter) converts these to mineral forms and increases their concentration relative to the starting material. The two biochars investigated in this work are typical examples of this situation.

The enhancement of plant protein productions clearly distinguishes the TP treatment. Plant productivity is not the only issue involving nitrogen. Its utilization in agriculture is another big environmental issue. After human perturbation of the global carbon (C) cycle, anthropogenic alteration of global N turnover is the second most important driver of global change [18]. Nitrogen is also the primary growth-limiting nutrient in many terrestrial ecosystems, and therefore is a fundamentally important component of ecosystem function. Perturbation of N turnover is to a large extent driven by the increased dependence of modern agriculture on the production and use of synthetic N fertilizers in crop production. About 85–90 million tonnes of nitrogenous fertilizers are added annually to the soil worldwide [25]. Nitrogen is one of the most expensive nutrients to supply, and commercial fertilizers represent the major cost in plant production. Furthermore, there is serious concern regarding nitrogen loss in the field, giving rise to soil and water pollution. Incomplete capture and poor conversion of nitrogen fertilizer also causes global warming through emissions of nitrous oxide. Lowering fertilizer input and breeding plants with better nitrogen uptake efficiency is one of the main goals of research on plant nutrition. In this context, the use of non-processed plant residues such as TP to promote N uptake by growing plants is the most economical and the eco-compatible mean to promote natural C and N turnover.

The results of this work on radish and those of the previous work [8] on bean plants, performed with the same TP, SOL and INS products, are a direct proof that the plant N uptake is a function of soil parameters as well as plant parameters. Thus the superior performance of TP in radish cultivation cannot be generalized for all plants. These results indicate that the issue of non-processed *vs.* processed biowaste for agriculture cannot be settled in a simple way. Complex plants, soil, climate conditions, fertilization practices and chemical form of applied nutrients interrelationships come into play. Species-specific nutrient use strategy of plants and their responses to resource amendment are important factors. There are many different types of biochar, fermented biowastes and non-processed agriculture residues potentially available, each with different short and/or long-term effects on plant growth. These materials could be the basis for blends, mixes and agro-chemical products. The best option appears that processed and non-processed wastes were used in dedicated formulations for specific cultivations and purposes. In this perspective, it would be much more interesting also to handle the same biowaste with both hydrolysis and pyrolysis processes. This would allow a comparison of the effects of products obtained from the same biomass with the two different processes. Given the economic and environmental relevance associated with the use of the investigated materials, the results of the present work offer an intriguing scope for further worthwhile investigation to understand the reasons of the differences of the observed effects and, hopefully, develop new products and practical guides for their best use in agriculture.

## 3. Experimental Section

### 3.1. Products Used in Cultivation Trials

Tomato plants (*Lycopersicon esculentum* Cv. *Naomi F1*) were grown in a greenhouse at the Angelo Zocco farm in Rosolini (SR), Italy. At the end of the crop harvesting season the exhausted plants were pulled out of the soil, roughly ground to 5–10 mm size on site and transported to the Studio Chiono pilot plant in Rivarolo Cavanese (TO), Italy. The material (TP) was further ground down to <0.5 mm particle size by use of Cimma, Pavia SF75 mill for use in the plant growth trials. An aliquot of the fine TP was reacted 4 h with KOH solution at pH 13, 60 °C and 4 *v*/*w* water/solid ratio. The liquid/solid hydrolysate mix was allowed to settle to separate the supernatant liquid phase containing the soluble substances (SOL) from the insoluble residue (INS). The recovered liquid phase was circulated at 40 L/h flow rate through a 5 kD off polysulphone ultrafiltration membrane operating with tangential flow at 7 bar inlet and 4.5 bar outlet pressure to yield a retentate with 5%–10% dry SOL content. The INS residue was washed once with fresh water at 4 *v*/*w* added water/solid ratio. The recovered ultrafiltration retentate and the humid INS residue were allowed to concentrate and/or dry in a ventilated oven at 60 °C to yield the final SOL and NS products which were used in the pot cultivation trials. The pristine TP organic matter was recovered 30% as SOL and the rest as INS. Further details on TP, SOL and INS preparation and characterization [8] and on miscanthus [10] and poultry litter [11] biochar preparation by pyrolysis at 650 °C and characterization are as previously reported.

### 3.2. Plant Growth Trials

*Raphanus sativu*s (Donar F1 by Syngenta) was sawn into nursery trays and afterward transplanted at three leaves stage into 8 × 8 × 9 cm pots.

Control plants were grown on a commercial substrate (Evergreen by Turco snc, Albenga, SV, Italy. The treatments were carried out by mechanically mixing 140 g substrate with 1.9 g TP, 1.0 g SOL, 2.6 g INS, 1.7 g BIOP, and 1.7 g BIOM respectively. One plant per pot was immediately transplanted and the pots were placed in a climatic cell at 25 ± 1 °C with a 16/8 h photoperiod. After 40 days the shoots, roots and leaves were harvested, weighted and stored at −80 °C.

### 3.3. Determination of Chlorophylls and Carotenoids Content

The determination of chlorophyll *a* and *b* and of carotenoids was performed on each plant by extraction of 300 mg fresh foliar tissue ground in liquid nitrogen with 10 mL 96% *v*/*v* ethanol. The samples were kept in the dark for 2 days at 4 °C, and the extracts were filtered and then analyzed by spectrophotometry using a Hitachi U-2000 spectrophotometer (Hitachi Group, Milano, Italy). The absorbance readings were performed at 665 nm for chlorophyll *a*, at 649 nm for chlorophyll *b*, and at 470 nm for total carotene. Chlorophyll *a*, *b* and total carotenoid concentrations were calculated as previously reported [8].

### 3.4. Determination of Soluble Proteins

Plant tissues were ground in liquid N_2_, with 1 mL/g of 0.1 M Na-P buffer, pH 7. The homogenates were centrifuged at 15,000× *g* for 15 min. All steps were performed at 4 °C. The protein concentration was determined in the supernatant as previously reported [8], using a UV/Vis spectrophotometer (Hitachi 2000, Hitachi Group, Milano, Italy) at 595 nm.

### 3.5. Physico-Chemical Characterization

C and N content were measured by elemental analysis of 0.5 mm sieved samples. Ash content was measured by incineration at 650 °C for 4 h. The total P content was determined colorimetrically (phosphomolybdic complex), after nitric-perchloric acid digestion. The soluble P content was determined colorimetrically after bicarbonate extraction.

For the total metals and silicium contents determination the ash of the biochars obtained at 550 °C for 12 h were treated with lithium metaborate at 950 °C for 30 min. The resulting material was then diluted in 10% (*v*/*v*) nitric acid and analysed by inductive coupled plasma atomic emission spectroscopy (ICP-OES) with a Perkin Elmer (Milano, Italy) Optima 7000 DV instrument.

The metals and silicium in TP, SOL and INS were mineralized by boiling in regal water for 12 h. The solution was then analysed by ICP-OES. Soluble metals concentration was determined by ICP-OES after 72 h water extraction. Silicium soluble concentration was determined by ICP-OES after 5 h extraction with 0.01 M CaCl_2_.

### 3.6. Statistical Treatment of Data

One-way ANOVA (*p* < 0.05) followed by the Tukey test for Multiple Comparison Procedures was performed using GraphPad Prism version 4.00 for Windows, GraphPad Software, San Diego, CA, USA, www.graphpad.com.

## 4. Conclusions

For origin and/or fabrication, the investigated TP, SOL, INS, BIOP and BIOM offer a range of materials differing widely for chemical nature and process energy consumption. The lowest energy consumption TP have been found the most effective in promoting radish plant N assimilation. Although the chemical composition of both the organic and the mineral fraction has been characterized, the experimental data do not allow the establishment of a relationship between the amounts of C, N, K, P and other mineral elements contributing to the cultivation medium by the above added materials and the plant performance indexes. The data certainly offer scope for further work by researchers in multidisciplinary fields. The reasons for the superior effects exhibited by TP are not yet clear. Nevertheless, the results suggest that, depending upon objectives and circumstances, a most economical and eco-friendly fertilization practice may be to recycle non-processed agriculture residues to soil. These can supply all necessary organic and mineral elements for plant growth. At the same time, such practice does not require investment and operational costs for biowaste processing. Thus, on-site recycling of non-processed agriculture residues, to the same soil where they are produced, implies benefits for both agriculture and waste management.
